# Inter-species cell detection - datasets on pulmonary hemosiderophages in equine, human and feline specimens

**DOI:** 10.1038/s41597-022-01389-0

**Published:** 2022-06-03

**Authors:** Christian Marzahl, Jenny Hill, Jason Stayt, Dorothee Bienzle, Lutz Welker, Frauke Wilm, Jörn Voigt, Marc Aubreville, Andreas Maier, Robert Klopfleisch, Katharina Breininger, Christof A. Bertram

**Affiliations:** 1 Pattern Recognition Lab, Computer Science, Friedrich-Alexander-Universität Erlangen-Nürnberg, Erlangen, Germany; 2 Research and Development, EUROIMMUN Medizinische Labordiagnostika AG, Lübeck, Germany; 3 VetPath Laboratory Services, Ascot, Western Australia; 4 Department of Pathobiology, OntarioVeterinary College, University of Guelph, Guelph, ON Canada; 5 Cytology Laboratory, Lungen Clinic Grosshansdorf, Großhansdorf, Germany; 6 Technische Hochschule Ingolstadt, Ingolstadt, Germany; 7 Institute of Veterinary Pathology, Freie Universität Berlin, Berlin, Germany; 8 Department of Artifical Intelligence in Biomedical Engineering, Friedrich-Alexander-Universität Erlangen-Nürnberg, Erlangen, Germany; 9 Institute of Pathology, University of Veterinary Medicine, Vienna, Austria

**Keywords:** Cellular imaging, Computational science, Respiratory tract diseases

## Abstract

Pulmonary hemorrhage (P-Hem) occurs among multiple species and can have various causes. Cytology of bronchoalveolar lavage fluid (BALF) using a 5-tier scoring system of alveolar macrophages based on their hemosiderin content is considered the most sensitive diagnostic method. We introduce a novel, fully annotated multi-species P-Hem dataset, which consists of 74 cytology whole slide images (WSIs) with equine, feline and human samples. To create this high-quality and high-quantity dataset, we developed an annotation pipeline combining human expertise with deep learning and data visualisation techniques. We applied a deep learning-based object detection approach trained on 17 expertly annotated equine WSIs, to the remaining 39 equine, 12 human and 7 feline WSIs. The resulting annotations were semi-automatically screened for errors on multiple types of specialised annotation maps and finally reviewed by a trained pathologist. Our dataset contains a total of 297,383 hemosiderophages classified into five grades. It is one of the largest publicly available WSIs datasets with respect to the number of annotations, the scanned area and the number of species covered.

## Abstract (summary)

**Table:** 

Measurement(s)	Hemosiderin-Laden Macrophage • Hemosiderin-Laden Macrophage
Technology Type(s)	machine learning • visual observation method
Sample Characteristic - Organism	Homo sapiens • Felinae • Equus caballus

## Background & Summary

In recent years, deep learning has revolutionised microscopy-based image recognition. Outstanding results can be achieved in well-defined tasks under the condition that sufficient high-quality datasets are available^1–3^. For certain species and/or certain pathologies, however, available data may be sparse. Approaches such as transfer learning and domain adaptation provide the possibility to develop algorithms that generalise across species although they come with their own challenges and limitations^2^. The generalised applicability of deep learning models between species could offer enormous scientific and economic value. For domains that lack appropriate training data, for example due to data protection and privacy restrictions, approaches that allow for this transferability may especially be useful in the context of animal models for human diseases.

To be able to develop, investigate and apply these algorithms, suitable cross-species datasets have to be available. The dataset described in this work aims to tackle several gaps present in currently available datasets. Firstly, whereas there are a couple of highly domain and target specific whole slide image (WSI) datasets publicly available for tissue^1–3^, to the authors’ knowledge none for cytologic research questions. Secondly, no publicly available dataset provides annotated WSIs from multiple species for the same pathology. Finally, as shown in our previous publication^4,5^, there is a high inter- and intra-observer variability for grading pulmonary hemosiderophages, which can be reduced by algorithmically supporting experts during labelling. For the development of these algorithms, large high-quality datasets are required, which is a further motivation for this publication.

In the following sections, we will describe the creation of a novel multi-species Pulmonary hemorrhage (P-Hem) WSI dataset. P-Hem describes repeated bleeding into the lung and can have a broad range of causes like congestive heart failure, leukaemia, physical exercise, or autoimmune disorders^6–11^ with possible life-threatening consequences^12^. In sport horses, a specific disease entity called exercise-induced pulmonary hemorrhage (EIPH) has very high incidences and may lead to reduced athletic performance^9,13,14^. This disease has therefore high relevance for the equine sport industry and has been used as an animal model for human P-Hem^15^. P-Hem is often diagnosed by cytologic examination of pulmonary fluid (bronchoalveolar lavage fluid (BALF)) with quantification of hemosiderin content in alveolar macrophages^7,16^. In chronic bleeding, macrophages (hemosiderophages) degrade red blood cells into hemosiderin, which is a protein complex containing iron. Usually special stains for iron, such as Prussian Blue or modified Turnbull’s Blue (Quincke reaction), are used to highlight the hemosiderin concentration in alveolar macrophages. For diagnosis of P-Hem in humans, a 5-tier grading system has been developed by Golde *et al*.^7^ and Doucet and Viel^17^ have adapted this system for EIPH in horses. Hooi *et al*.^11^ have recently described a similar scoring system for cats.

For the creation of this novel dataset, we digitised and fully annotated 55 equine, seven feline and 12 human BALF samples with a total of 297,383 manually verified macrophage annotations in form of bounding boxes. To improve labelling efficiency and data quality, we applied expert-algorithm cooperation in the following manner. Firstly, we incorporated a publicly available pre-trained EIPH model^4^ for equine WSI grading to our multi-species dataset resulting in 585,600 candidate annotations. Secondly, visualisation and clustering techniques were applied to semi-automatically remove 45,944 false positive annotations. Afterwards, a trained pathologist (C. A. B.) performed a screening and reviewed the complete dataset which left 303,289 hemosiderophages. As a final validation step, the hemosiderophages were arranged and presented according to their grade and conclusively checked by the same trained pathologist (C. A. B.) resulting in a total of 297,383 manually verified annotated hemosiderophages.

**Fig. 1:**
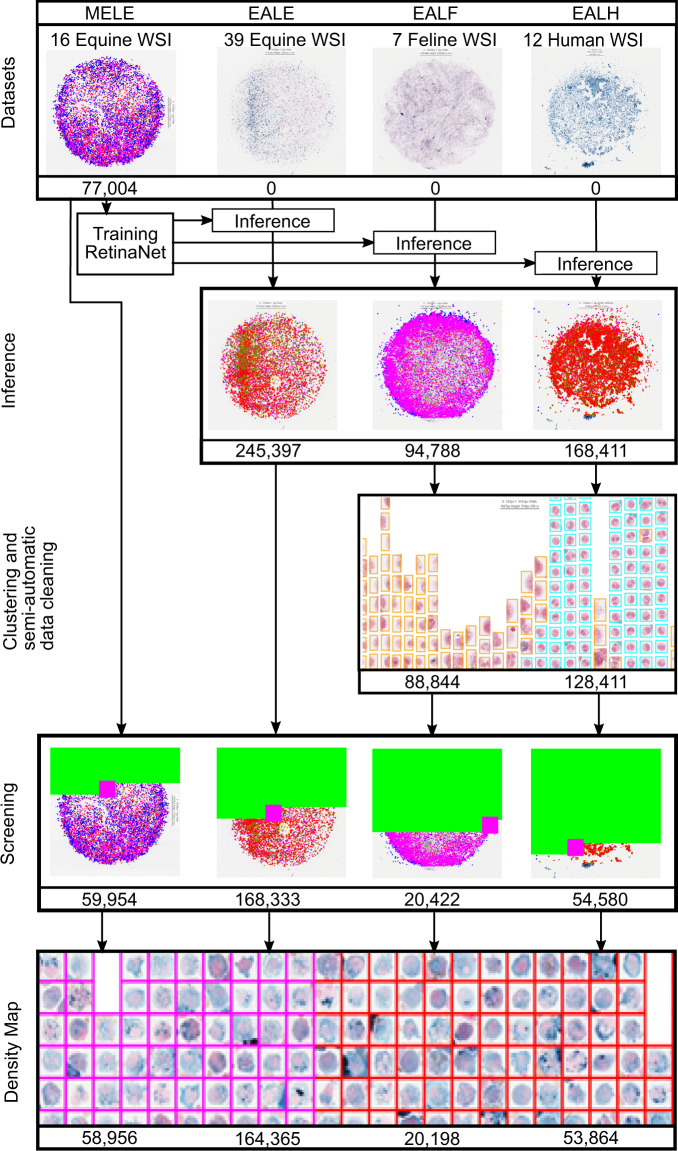
Overview of the macrophage annotation and validation pipeline: The publicly available RetinaNet object-detection model trained on equine slides^4^ is used to perform inference on the unannotated slides, followed by a semi-automatic clustering step which clusters cells by size. Error-prone cells are highlighted and can then be efficiently deleted by a human expert. Afterwards, a human expert screens all WSI to increase the dataset consistency. Finally, a regression-based clustering system is applied to support experts searching for misclassifications of the hemosiderin grade.

As a result of this expert-algorithm pipeline which is visualised in Fig. 1), we present the largest publicly available fully-annotated multi-species cytology WSI dataset to date. Our dataset provides researchers with unprecedented opportunities to develop new inter-species algorithms and can help to overcome domain adaptation limitations. We evaluated the quality of the dataset by conducting a species-wise 3 × 3 cross validation and performed an ablation study to estimate how many annotated WSIs are needed to adapt to new species.

## Methods

The following section describes the sample collection, staining and digitisation procedure followed by our annotation processing pipeline. The balf samples of the three species were collected at different institutes for routine diagnostic evaluation of respiratory disease. Therefore, no animal was harmed for the creation of this dataset. Individual case histories were not considered in the present study and all samples we received were anonymised by the providing laboratory. Approval for use of animal specimens was given by the State Office of Health and Social Affairs of Berlin (approval ID: StN 011/20) and for human samples by the University of Lübeck (approval ID: AZ 19–428). The 74 cytological slides were prepared by cytocentrifugation and stained for iron content with Prussian Blue (n = 37) or modified Turnbull’s Blue using the Quincke reaction (n = 37). Both staining methods result in similar insoluble blue pigments^18^ and therefore similar hemosiderophages appearances. Digitisation of the glass slide was performed using a linear scanner (Aperio ScanScope CS2, Leica Biosystems, Germany) at a magnification of 400× (resolution: $0.25\frac{\mu m}{px}$). To be as consistent as possible in the data pre-processing phase, all samples were stained and digitised in the same laboratory (Institute of Veterinary Pathology, FU Berlin).

### Equine datasets

Fifty-seven equine samples were prospectively collected at the VetPath Laboratory Services (Australia) from 29 BALFs samples of 25 horses with clinical signs of lower respiratory tract disease. Samples were prepared by cytocentrifugation (CYTOPRO 7620, Wescor Inc, Logan, UT, USA) at 510 × g for 3 minutes using a variable volume of BALFs depending on cellular density. Subsequently unstained slides were shipped to the FU Berlin, Germany, and stained with both staining methods and digitalized as described above.

#### Manually expert labelled equine (MELE) dataset

A preliminary dataset using 17 equine WSIs was developed for a previous publication^4^ and revised for this publication. Initially, these slides were fully annotated by one expert (C. A. B.) with the open source software SlideRunner^19^ in a two stage process. First all macrophages/hemosiderophages were annotated by screening the WSIs and afterwards cell annotations were assigned a corresponding grade. From these 17 WSIs, 16 were added to this publication and one was removed due to a significant fungal contamination (>1% of the cells) in the Turnbull’s blue staining, resulting in 10 Prussian Blue and 6 Turnbull’s Blue samples from 16 horses. Subsequently (for this publication), the same expert (C. A. B.) modified this dataset by a second screening process and review of the grades with the help of density maps (see section Density map). In the following, we will refer to this dataset as manually expert labelled equine (MELE) dataset.

#### Expert-algorithm labelled equine (EALE) dataset

For the creation of the expert-algorithm labelled equine (EALE) dataset, we used 39 additional WSIs from 26 horses. A detailed overview regarding the dataset’s meta-data can be accessed at the supplementary Table images_meta_data.csv. The samples were prepared at the same laboratory as the MELE dataset and were processed according to the same protocol. The dataset consists of 18 Prussian Blue and 21 Turnbull’s Blue samples. The database was created by interference of the WSIs with an algorithm developed on the initial dataset (MELE) and multiple steps of quality control (Clustering, Screening, Density maps) as summarised in Fig. 1.

### Expert-algorithm labelled feline (EALF) dataset

Seven feline samples were retrospectively obtained from the study by Hooi *et al*.^11^, which was designed to evaluate the presence of hemosiderophages in feline BALF samples. Samples were initially prepared by cytocentrifugation and stained with Wright’s stain^11^. For this study specimens were de-stained and re-stained with Turnbull’s Blue. The re-staining of WSIs is assumed to have a negligible effect on dataset creation in light of the applied expert-algorithm collaboration pipeline which can correct for algorithmic confusions due the lower quality of the input data^4,5^. Labels were created by interference and a multi-step quality control (Clustering, Screening, Density maps). In the following, we will refer to this dataset as expert-algorithm labelled feline (EALF).

### Expert-algorithm labelled human (EALH) dataset

The samples were collected by a BALF procedure using local anaesthesia bronchoscopy. In all cases humans did not undergo any steroid or other immunoregulatory therapy. After the volume of recovered BALF had been assessed, the fluid was filtered through a layer of sterile gauze, centrifuged (15 min, 4 °C, 65 × g) and resuspended. Total cell counts were assessed in a Neubauer chamber and viability was determined by trypan blue exclusion. Each cytospin slide was prepared from BALF with 50,000 cells (600 cpm, 15 min; Heraeus Sepatech Omnifuge 2.0 RS, Hanau, Germany). Following staining with May-Grünwald-Giemsa and HEMATOGNOST Fe® SIGMA routine cytological examination were performed to confirm P-Hem due to different underlying diseases. Supplementary preparations were made of 12 cases with proven P-Hem and unstained specimens were subsequently send to FU Berlin and three stained with Turnbull’s blue and nine with Prussian Blue. In the following, we will refer to this dataset as expert-algorithm labelled human (EALH) dataset.

### Labelling and visualisation platform

To create this multi-species WSI dataset, we used the open source online platform EXACT^20^, which was specifically modified for this project. The software supports the creation of this dataset with multiple features which we will briefly summarise in the following section. Manual WSI annotations are supported by a special screening mode, which allows for systematic screening of slides in a user-defined magnification while saving the progress per expert and therefore allowing to conveniently resume the work at a later point in time. Furthermore, a bounding box annotation process is streamlined by a single-click annotation mode which incorporates the average hemosiderophages size and therefore minimises the need to further adjust the bounding box to the cell size. Annotation versioning supports the tracking of changes and provides detailed and reproducible insights into the development process of datasets.

### Inter-species inference from a pre-trained model

At the time of dataset development, no annotations for feline or human P-Hem slides were publicly available, which resulted in limited options to perform transfer learning-based methods. Therefore, we directly applied the publicly available^4^ equine P-Hem deep learning model trained on the MELE dataset to the WSIs of the EALH, EALF and EALE dataset (Fig. 1 Inference). The equine deep learning model uses a custom RetinaNet-model^4,21^ optimised for hemosiderophage WSI detection. The model was trained with the Adam optimiser on 14 fully annotated WSI from the MELE dataset until convergence was reached by a maximal learning rate schedule of 0.01. The model was validated on three remaining fully annotated WSIs from the same dataset. As described in section MELE, for this publication, we excluded one slide due to considerable fungal contamination, resulting in 16 MELE slides.

Inference on the 58 unannotated WSIs of the EALE, EALF, EALH datasets took on average 120 seconds per WSI on an NVIDIA Quadro P5000 graphics card. To minimise the probability of missing hemosiderophages, we applied a classification probability threshold of 0.35 to all slides to obtain a highly sensitive and less specific model resulting in 585,600 macrophage/hemosiderophage candidate annotations.

### Semi-automatic data cleaning via customised clustering

The accuracy of deep learning models depends on multiple factors, which are oftentimes difficult to control. One influencing factor, that may lead to varying results, is the quality of the source dataset, which, in turn, strongly depends on various pre-analytic steps such as image acquisition. Additionally, the label quality used for training deep learning models has a strong influence on the final performance, and for P-Hem grading a high inter- and intra-observer variability has previously been described^4,5^. Special stains for iron are ideal to quantify the intracytoplasmatic hemosiderin content (stained as blue pigment), but introduce considerable difficulties in differentiation of different cell types due to the weak staining of cellular components. One additional aspect is the domain shift between species, which might manifests in altered cell morphology and texture compared to the source domain (i.e., equine tissue). An example for this domain shift artefacts is the reduced performance of the initial algorithm on the feline samples due to false-positive detections of granulocytes or multiple bounding box predictions per cell.

To minimise the effect of the above-described implications on this dataset, we established the following semi-automatic pipeline. Firstly, all cell patches of a slide were copied into a new image on the EXACT server and sorted by width in ascending order on the x-axis (Fig. 1 3rd row, Clustering). Predictions were grouped by width-to-hight-ratio of the bounding box in a annotation map. Thereby a human expert could remove obvious false positive predictions (small cell types and non-maximum-suppression artefacts) using the web interface. This is implemented by drawing a rectangle with a computer mouse around groups of cells to delete them from the dataset. Aforementioned size-based visualisation also allowed the efficient re-labelling of false-positive granulocytes in feline samples due to their significant smaller cell size compared to macrophages. The semi-automatic data cleaning step removed 17.45% of the cells created at the inference step.

### Experts screening

For labelling data, expert-algorithm collaboration is considered suitable for creating high quality datasets^5^. Diligent expert review of algorithmic predictions is indispensable, especially for WSIs that may potentially exhibit a significant domain shift to the initial training data. To keep the screening process as consistent as possible, the same veterinary pathologist (C. A. B.) performed all annotation tasks. To enable an efficient validation of all algorithm-created annotations across the WSIs, we used the screening mode provided by the EXACT software. With this mode, it is possible to check a WSI patch by patch and correct errors on a user-selected magnification. An overlap of 15% per patch is applied, and the expert’s progress is saved automatically (Fig. 1 Screening). In this screening step, the expert removed 44.8% of the automatically detected cells (236,367) and introduced 560 new hemosiderophages on 51,110 patches. These numbers are in line with the high sensitivity and low specificity expected from setting low cutoff values for algorithmic predictions. Similar to the screening of the computer aided annotations, the original manual annotations from the equine MELE dataset initially developed for a previous publication^4^ were reviewed. Here, the expert deleted 17,050 of the 77,004 annotations (22.1%) and introduced 30 new annotations. Deletion of such a high number of manual and algorithmic labels was mostly attributed to the difficulty of classifying different cell types (macrophages versus other cell types) with the special iron stain. Clear identification of macrophages (including hemosiderophages) in BALF is largely based on morphology of the cell nucleus which is, however, only very weakly highlighted with iron stains. Cellular size and shape alone are only vague cell classification criteria. We have noticed that the task of distinguishing hemosiderophages against neutrophils may be complicated by the positive iron staining of both cell types. While the initial manual labelling of the MELE had a high sensitivity for labelling hemosiderophages, its re-evaluation suggested that many neutrophils had been wrongly annotated. During expert screening, unambiguous non-macrophagic cells, especially cells with a small cell size, were deleted, however this had no influence on the overall hemosiderin score of the respective WSI.

### Density map

**Fig. 2:**
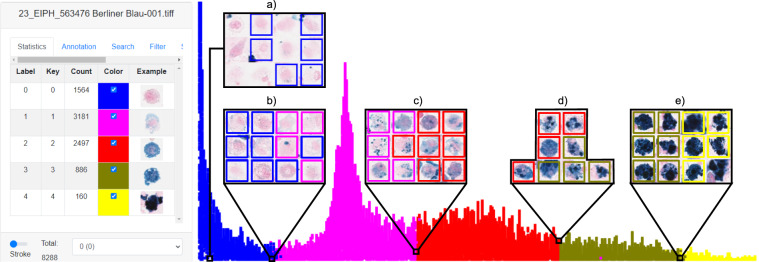
Left: Statistics on the density map at the EXACT user interface. Right: Visualisation of a density map which was screened by an expert for mislabelled cells (especially regarding the label class). Subfigure a) displays six manually deleted annotations. Visualisations b) to e) show the border region between two grades, with the first two columns representing the lower grade and the last two the upper grade, which were sometimes corrected by the expert as visualized by the different color of the bounding box.

Initially, all hemosiderophages were classified into discrete grades from zero to four depending on their hemosiderin concentration, both for computer-aided annotations for the EALE, EALF, EALH dataset and the expert-created annotations for the MELE dataset. However, the hemosiderophages hemosiderin absorption is a continuous process which is only mapped to a discrete grading system. This can lead to inconsistent classification between neighbouring grades as previously described by Marzahl *et al*.^4^. To overcome this limitation, we utilised the provided cell-based regression approach^4^ to assign a continuous grade between zero and four to each hemosiderophage. Afterwards, we created a new image-map where the hemosiderophages were arranged in an ascending order along the x-axis according to their hemosiderin score. These novel image-maps were created for each WSI individually and reviewed by the same trained pathologist (C. A. B.) to make the process of identifying mislabelled cells on the border between two grades (Fig. 2 Density Map) as consistent as possible. On the density maps the expert changed the grade of 38,799 (13.04% Up: 13,591 Down: 25,208) annotations from which 99.92% were changes within one grade. The density maps also provided another opportunity to review the cell type of the annotations, which were deleted in 5,906 (1.95%) instances.

## Data Records

We provide the 55 equine, 12 human and seven feline original WSIs in the Aperio SVS format without any identification properties publicly available on figshare^22^. Alongside, we supply all hemosiderophage annotations after each of the four processing steps (Inference, Cluster, Screening, DensityMap) as comma-separated files for easy access, as binary files which are compatible to our training and evaluation pipelines, and in the sqlite format for SlideRunner^19^. Each annotation provides the following information:

- The annotation source slide name
- A universally unique identifier (UUID)
- The absolute bounding box coordinates (x1,y1,x2,y2) on the WSI
- The EIPH grade in a discrete range from zero to four

Additionally, we provide a Docker build with all packages installed to download the WSIs and annotations for reproducing our experiments. Table 1 gives an overview of the dataset’s meta-data. A detailed per-image statistic can be examined in the supplementary Table images_meta_data.xlsx. The dataset column distinguishes between MELE, EALE, EALF and EALH datasets. The version column indicates the processing steps (Inference, Cluster, Screening, DensityMap) to which the following statistical data refer. The EIPH score was calculated by the method of Doucet and Viel^17^.

**Table 1:** Overview of the dataset meta-data, including the species, the dataset name, the number of slides, the version of the post-processing refinement step (**I**nference, **C**luster, **S**creening, **D**ensityMap) and the number of labels per hemosiderin score.

species	dataset	slides	Version	Total Cells	Score	Count of Cells by Grade				
						0	1	2	3	4
Equine	MELE	16	MELE	77,004	102	29,017	26,810	13,178	6,577	1,422
			S	59,954	112	19,733	21,545	11,442	5,963	1,271
			D	58,956	109	19,246	21,595	11,829	5,552	734
	EALE	39	I	245,397	95	97,904	80,715	47,789	17,437	1,552
			S	168,333	108	54,432	60,189	39,316	13,404	992
			D	164,365	101	51,797	67,798	36,339	7,810	621
Human	EALH	12	I	168,411	133	31,035	64,833	58,320	12,776	1,447
			C	128,012	133	21,532	53,704	42,553	8,932	1,291
			S	54,580	156	47,26	20,688	23,323	5,090	753
			D	53,864	156	43,84	18,357	26,563	4,433	127
Feline	EALF	7	I	94,788	38	58,879	35,659	122	8	120
			C	88,848	38	54,867	33,868	103	5	5
			S	20,422	33	13,631	6,747	41	2	1
			D	20,198	45	11,124	9,039	35	0	0
Total	SDATA	74	I	585,600	98	216,835	208,017	119,409	36,798	4,541
			S	303,289	113	92,522	109,169	74,122	24,459	3,017
			D	297,383	109	86,551	116,789	74,766	17,795	1,482

In total, the expert screened 51,110 patches on 74 WSIs from three species which covers a total area of 5,196.17 *mm*^2^. This resulted in 297,383 annotated macrophages/hemosiderophages, making this the largest published multi-species dataset of macrophages/hemosiderophages and one of the largest cytology WSI datasets in general.

## Technical Validation

To gain a deeper understanding of the data and to establish a baseline for future studies, we conducted multiple experiments. Firstly, during the screening phase, we noticed that the expert (C. A. B.) deleted a high number of his own manually created annotations from the dataset of our previous work (MELE dataset). Furthermore, our deep learning method, which was trained on these initial annotations, also introduced many false positive annotations even at conservative thresholds. This effect was amplified by the decision to configure the model with a relatively high sensitivity in order to miss as few cells as possible. The observation that the initial object detection model was configured to have a high sensitivity (and therefore a low specificity) is backed by the statistics that only 560 new hemosiderophages were introduced in the screening phase of the dataset development (EALH, EALF, EALE) compared to 229,054 deleted cells. The combination of these effects caused the manual deletion of large quantities of annotations as shown in Table 1. To quantify and compensate for this effect, in the following first experiment, we investigated if the trained deep learning model can be efficiently adapted to this change in annotation behaviour by retraining on the updated annotations from the MELE dataset created for this publication. In a second experiment, we evaluated inter-species domain transfer and performed an inter-species cross-validation study. This experiment is followed by an ablation study to estimate the quantities of annotations needed to train an accurate EIPH object detector. To evaluate the object detection performance of the models trained in our experiments, we used the mAP metric introduced in the 2007 PASCAL VOC challenge^23^.

### Reevaluation of the inference step

To investigate whether and how efficient the deep learning model can adapt to the changed annotation behaviour, we trained models with the initial and reviewed MELE dataset and optimised thresholds for the different datasets individually. To make the results comparable to the initial publication^4^, we used the original 17 slides, including the slide with fungal contamination. We applied the customised RetinaNet architecture with a ResNet-18 pre-trained on ImageNet. The network was trained with the Adam optimiser using a maximal learning rate of 0.001 until the validation loss started to increase. As a metric to quantify how effective the deep-learning model adapted to the new annotations we calculated the mAP score with an intersection over union (IoU) >0.5 and compared total cell numbers. The mAP score increased with the new annotations by 5 percent from 0.66 to 0.71 compared to the object detection results reported in earlier works. This indicates that the experts annotations are more consistent. The optimal threshold calculated on the validation set for equine samples increased from 0.35 to 0.65 and for humane and feline slides from 0.35 to 0.80. The total number of detections decreased from originally 585,600 to 301,109 (ground truth 297,383) while the number of false negatives increased from 560 to 7,351 according to the final dataset. In conclusion the deep-learning model is able to adapt to new annotation behaviours and a stronger focus on finding optimal thresholds could lead to decreased manual interactions but introduces the risk of overlooking false-negative annotations.

### Inter-species domain adaptation

**Fig. 3:**
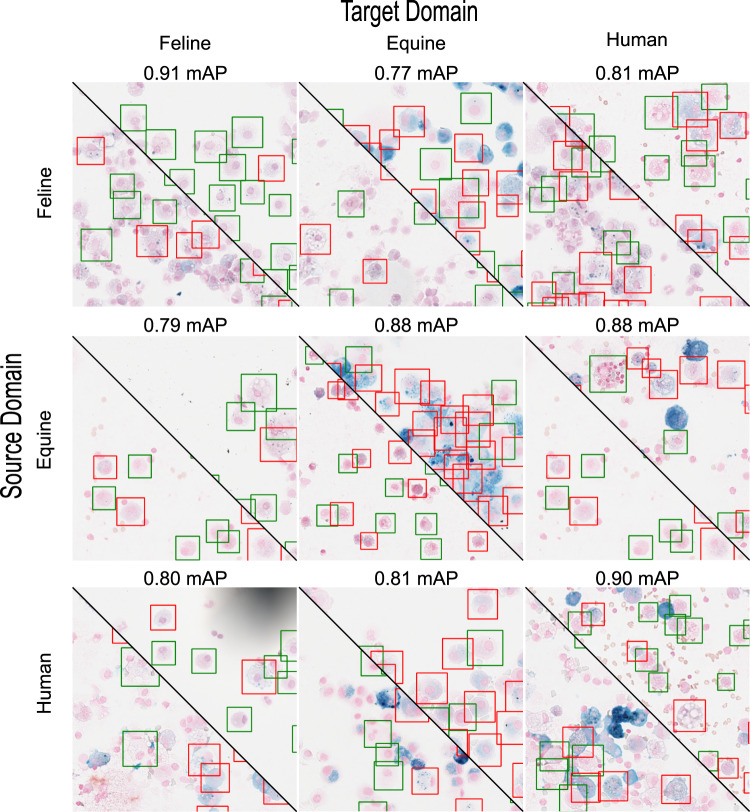
Each of the nine figures show on the left the species source training domain and on the top the species target domain with the obtained mAP. Green bounding boxes represent grade zero hemosiderophages while red show grade one.

As shown by Bullone *et al*.^15^, equines can be used to better understand human asthma on an immunopathological level. To support scientific research in this direction, the use of machine learning models across species is of great scientific and economic importance. To investigate the potential and limitations of transferability across different species, we have carried out a 3 × 3 cross-validation in which we trained on one species and validated on all other species separately. To support the comparability of the results across the species with their varying amount of available WSIs and to keep the computational effort within reasonable limits, we decided to use only five WSIs for training and two other WSIs for validation (See Table 2). This is further motivated by availability of only 7 feline WSIs. For the other two species, the training and validation subset was selected by using the seven most balanced slides with respect to the number of grade zero and one macrophages/hemosiderophages (see Table 2). We used this WSI sampling strategy to minimise the effect of an imbalanced dataset which could negatively impact the transferability study. Due to the circumstance that feline WSIs only contain hemosiderophages with the grades zero and one we only used these two classes for the cross-validation for all species and reason that the transferability of these two classes can be generalised to the remaining classes. Example patches and results from this cross-validation experiment are visualised in Fig. 3. The experiment achieves best results if the source is equal to the target domain with an mAP value of 0.90 (Equine 0.88, Human 0.90, Feline 0.91). The training on equine slides resulted in an mAP of 0.88 on human data which indicates that a domain transfer without adaptions to the deep learning model might be possible. Further studies need to show if this algorithms can be used for specific disease of humans such as COVID-19^24^. When the source domain is human or feline, the average inter-species mAP is 0.8 (min 0.77, max 0.81). Moreover, EIPH can also affect other species such as dogs^11^ and future studies may evaluate if the described domain transfer can be reproduced.

**Table 2:** The filenames of the five training and two validation slides (below the double line) per species used for the ablation and inter species cross-validation study.

File	Species	Count of Cells by Grade				
		0	1	2	3	4
15.svs	Equine	2884	3124	2138	733	12
22.svs	Equine	2147	2441	2025	899	69
30.svs	Equine	2815	2464	1523	606	26
19.svs	Equine	1197	1012	316	36	0
02.svs	Equine	2094	2560	1291	364	2
2707.svs	Human	1434	2954	1034	122	0
11480.svs	Human	391	1221	674	20	0
10080.svs	Human	284	3015	2375	342	9
10052.svs	Human	120	1739	3744	739	9
10120.svs	Human	112	2225	4656	1502	105
1.svs	Feline	1200	2287	22	1	0
6.svs	Feline	3009	895	0	0	0
14.svs	Feline	2393	495	2	0	0
13.svs	Feline	4663	810	2	0	0
2.svs	Feline	57	502	10	0	0
27.svs	Equine	1392	1359	364	93	1
17.svs	Equine	2725	2625	715	166	14
10227.svs	Human	1265	1943	324	5	0
2702.svs	Human	963	523	413	67	2
10.svs	Feline	412	371	4	1	0
12.svs	Feline	1897	1387	1	0	0

The slides have been selected and ordered according to their ratio of grade zero and one cells to represent a balanced sub-dataset.

### Ablation study

**Fig. 4:**
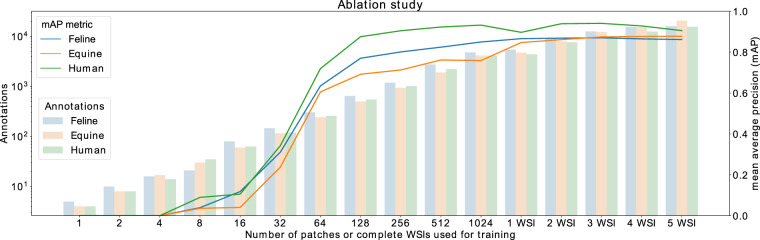
Results of the ablation study using our customised RetinaNet object detector on an increasing number of humane, equine and feline training patches of size 1024 × 1024 pixel from one WSI or up to five complete WSIs. The boxes represent the total number of hemosiderophages used for training in combination with the mAP graphs for each species.

Annotating WSIs manually is a laboursome and expensive task. Therefore, one of the most interesting questions in creating datasets and training deep learning models is the number of WSIs and annotations needed to reach a converging performance. To answer this question, we started training for each species separately on one uniquely sampled patch (size 1024 × 1024 pixels, number of annotations: mean = 6.19, SD = 3.74) from one slide and then doubled the number of patches from the same slide every time training reached convergences on the validation set. The training set was chosen to have a balanced number of grade zero and one hemosiderophages. The cell-covered area of each WSI contains on average 1,000 unique patches, therefore we continued the ablation study using up to five different WSIs for training after reaching the values of 1024 training patches on the first slide. To increase the comparability between our experiments, we used the same network, parameters, annotations and slides as described in the section domain adaptation. As visualised in Fig. 4, the performance of the model increased significantly independent of the species until 128 patches with around 1000 unique hemosiderophages and started to converge afterwards even if additional WSIs were introduced and the total number of annotations was increased up to twentyfold. As described above, to keep the experiments between species comparable, we only used grade one and two hemosiderophages and therefore reason that around five hundred cells per type are sufficient to reach convergence. To put this into perspective: 12 human samples contain only 127 grade four hemosiderophages, making the shown inter-species domain transfer highly valuable for creating deep learning models for human data. This is especially valid for P-Hem, which has a particularly high incidence in horses.

## Usage Notes

Due to multiple dependencies of our repository we provide a docker file to streamline the setup process and install all necessary packages for tracing and reproducing our results. The most prominent dependencies are: fast.ai^25^, a deep learning library which is build on PyTorch^26^, matplotlib^27^ for visualisation, object-detection-fastai with our custom RetinaNet implementation and OpenSlide^28^.

The repository is structured as follows: On the top level the “Download.ipynb” jupyter notebook will download all slides and annotations from figshare^22^ automatically. The folder Statistics contains notebooks which analyse the dataset annotations and general information about the slides. Inference contains code to train the described models and perform inference on slides. Regression trains the regression models to predict a continuous EIPH grade and is used for creating the density maps. Cluster contains code to create custom annotation maps and synchronise the generated images and annotations with EXACT.

## Data Availability

All code used in the experiments to generate results, plots and tables was written in Python and is available through our GitHub repository for EIPH analysis [https://github.com/ChristianMarzahl/EIPH_WSI/] in the folder SDATA and is referenced on Zenodo^29^.
